# Herb–Drug Interaction in Inflammatory Diseases: Review of Phytomedicine and Herbal Supplements

**DOI:** 10.3390/jcm11061567

**Published:** 2022-03-12

**Authors:** Annemarie Lippert, Bertold Renner

**Affiliations:** Institute of Clinical Pharmacology, Medical Faculty Carl Gustav Carus, Technische Universität Dresden, 01069 Dresden, Germany; bertold.renner@tu-dresden.de

**Keywords:** rheumatoid arthritis, inflammatory bowel disease, herbal medical products, complementary medicine, green tea, ginseng, cyclooxygenase inhibitor, NSAID, cyclosporine, methotrexate

## Abstract

Many people worldwide use plant preparations for medicinal purposes. Even in industrialized regions, such as Europe, where conventional therapies are accessible for the majority of patients, there is a growing interest in and usage of phytomedicine. Plant preparations are not only used as alternative treatment, but also combined with conventional drugs. These combinations deserve careful contemplation, as the complex mixtures of bioactive substances in plants show a potential for interactions. Induction of CYP enzymes and pGP by St John’s wort may be the most famous example, but there is much more to consider. In this review, we shed light on what is known about the interactions between botanicals and drugs, in order to make practitioners aware of potential drug-related problems. The main focus of the article is the treatment of inflammatory diseases, accompanied by plant preparations used in Europe. Several of the drugs we discuss here, as basal medication in chronic inflammatory diseases (e.g., methotrexate, janus kinase inhibitors), are also used as oral tumor therapeutics.

## 1. Introduction

Despite the dominance of medicinal products, with one, or few, chemically-defined active substance(s), in industrialized countries, herbal medicinal products remain popular, and their relevance even grows [1]. Surveys among pediatric, adult, and elderly patients in several countries reveal that between 15 and 45% of patients use herbal products for healthcare purposes, besides prescribed medicine [2,3,4,5,6]. The use of complementary herbal medicines and food supplements is particularly frequent among women and older people [7,8,9]. Surveys indicate that patients with chronic diseases use more supplements [8]. Many patients consider them “natural” and, thus, “less harmful” and having no, or fewer, side effects [6,10]. That may result in not considering them as medicine or medicinal products and missing to report them to health professionals, if not explicitly asked [5,6,10,11]. When patients use herbal medicinal products (HMP) or herbal supplements conjointly with conventional drugs, this bears the risk of unrecognized potential interactions.

The legal background of plant products for health purposes in the European Union is complex. The status ranks from herbal dietary supplements fall under food legislation and cosmetics to HMPs, with different kinds of market authorization. There are three kinds of HMPs in the European Union (regulated in Directive 2001/83/EC and its amendment, Directive 2004/24/EC [12]): (i) products based on “traditional use” with at least 30 years of documented usage and without known safety concerns, (ii) “well-established use”, and (iii) HMPs with a regular approval, based on preclinical and clinical data.

The Committee on Herbal Medicinal Products (HMPC), which belongs to the European Medicines Agency (EMA), develops monographs on herbal drugs and plant products, collecting evidence for “traditional” and “well-established” use, the latter requiring scientific evidence for beneficial effects, at least ten years of experience, and publications indicating safety. These monographs are considered when evaluating applications for market authorization. Products with documented “traditional use” or “well-established use” status gain market authorization through a facilitated process. A more detailed description of the regulatory environment in Europe, as well as its practical effects, can be found in the Bilia and Céu Costas review [13].

The complex regulation of HMPs and other botanicals reflects different levels of evidence, but products with less evidence cannot be regarded as generally inactive. It most often means that we have less knowledge of benefits, as well as risks. Food supplements are usually restricted to lower daily doses than medicinal products and, thus, considered safe. However, depending on individual susceptibility, with conditions such as age or renal impairment, medication, and consumer-driven dose changes, there still remains a risk for adverse effects and/or interactions. That means, for the purpose of risk assessment, all herbal products impartial of their legal status should be considered. This review aims to include plant derived products (extracts, powdered plant, and teas) used systemically for health improvement or treatment of disease, regardless of their legal status. Products for topical use are not in the focus of this review because of their lower potential for systemic drug interactions. Adverse effects, such as allergies, are more relevant for topic herbal products. Caution is advised for patients receiving phototoxic medication, as many plants, such as citrus species or chamomile, have phototoxic potential (reviewed in [14]). Classical homeopathic products are diluted and thus can usually be regarded as safe, in terms of interactions. However, practitioners should be aware of products with low potency (D6 and smaller) dilutions and undiluted original tinctures (often marked with Ø in the declaration). They can deliver plant metabolites in relevant amounts. The interaction potential of vitamins, minerals, or animal-originated products (such as glucosamine) is not part of this article. An overview can be found in [15,16,17,18].

There is extensive regional variation in the extent of herbal medicine usage and, more so, the preferred species based on tradition and availability. The interest in medical systems, such as Traditional Chinese Medicine or Ayurveda, is growing, but most of the plants used in these systems are still not common in western countries. It would go beyond the scope of this review to discuss popular medicinal plants of all world regions. Main focus of this article are plants originated from Europe or whose medicinal use is established in Europe and western countries.

Chronic inflammatory diseases, such as rheumatoid arthritis (RA) or chronic inflammatory bowel diseases (IBD), are characterized by complex immune mediated processes which maintain inflammation and destroy physical structures. Despite progress during the last decades, the pathophysiology is not completely understood and treatment is not always effective. Therapy regimens usually include long-term use of cytostatic-like or immune modulating drugs, often with small therapeutic windows and a high potential for adverse effects.

Other common diseases, such as degenerative osteoarthritis, show secondary inflammatory processes that intensify the progress. Though non-pharmacological treatments are the main therapy for these patients, application of analgetic drugs is common, often over years.

Many patients try to improve their condition using food supplements and/or complementary medicine [19]. This is more common for those with insufficient symptom control [19,20]. Herbs can change pharmacokinetic parameters by affecting absorption, distribution, metabolism, or elimination (ADME) or cause pharmacodynamic interactions. Most studies on herb–drug interactions focus on CYP enzymes or transporter molecules. Beyond that, additional toxic effects may occur. Several plants have been connected to drug-induced liver injury (DILI) or herb-induced liver injury (HILI). The risk is elevated by preexisting liver diseases, chronic alcohol consumption, and genetic CYP polymorphisms that either slow down metabolization of hepatotoxic drugs or accelerate the generation of toxic metabolites [21]. Women are more often severely affected by DILI than men [22].

This review aims to give an actual compilation of plants that are commonly popular among patients or could be used to treat inflammatory diseases and their interaction potential with important conventional drugs in this field. In an attempt for width, rheumatoid arthritis, chronic inflammatory bowel diseases, and osteoarthritis are used as examples to determine conventional medication and botanicals that could be used by patients.

## 2. Methods

To determine the plant species most commonly used for medicinal purposes in Europe, PubMed, and Web of Science were searched for surveys on HMP and herbal food supplement consumption. Papers published before 2000 were excluded to focus on more recent patient behavior. From the combined literature, a list of the most frequently occurring species was established.

Species that are used specifically to treat inflammatory diseases were determined from surveys among patients, preferably from European countries. Additionally, in an attempt to include the plants possibly used by patients, which do not appear in the scarce survey data, additional sources were used. That includes HMPC-monographs, treatment guidelines [23,24,25], and database searches (PubMed and Web of Science, search terms: herbal medicine, herbal supplement, rheumatoid arthritis, osteoarthritis, and chronic inflammatory bowel disease).

For the combined list of plant species, PubMed was searched for herb–drug interactions, without restrictions regarding the year of publication (search terms: scientific and English plant name, interaction). In vitro- and in vivo studies, case reports and meta-analyses were included. Review articles were searched for additional references. HMPC monographs, if existing, and their corresponding reference lists were consulted. Additionally, interaction checks were run on UpToDate (https://www.uptodate.com/drug-interactions/, accessed on 15 November 2021). The hepatotoxic potential of the prescription drugs are listed below and the herbal drugs, if available, were verified with LiverTox^®^ (https://www.ncbi.nlm.nih.gov/books/NBK547852/#IX-M, accessed on 22 October 2021) [26]. Search terms and results can be found in the supplement. Literature research was performed August until October 2021.

## 3. Assessment of Herbal Drugs and Their Interactions

### 3.1. Compilation of Plants

Most surveys on complementary medicine use or food supplement consumption ask for the use of botanicals, but not for the species used. Six general surveys, supplying data on plant species used for health improvement/medicinal purposes, conducted in European countries, were identified: a questionnaire completed by 271 outpatient clinic patients in the United Kingdom (UK) found 87 different species used in herbal supplements [27]. Garlic (taken by 31.9%), ginkgo (28.1%), echinacea (22.6%), evening primrose oil (17%), St. Johns wort (10.4%), ginseng (7%; without differentiation between Korean, American, and Siberian ginseng), *Aloe barbadensis* (7%), devils claw (5.6%), cranberry (4.8%), and saw palmetto (4.4%) were the ten plants most frequently reported. Djuv et al. conducted a questionnaire-based survey among patients ≥18 years on general practices in Norway [5]. Of 381 persons who completed the questionnaire, 44% used herbal products. Sixteen plant species were reported, of which bilberry, green tea, garlic, aloe vera, echinacea, cranberry, and ginger were taken by at least 10% of users. A survey with 8081 participants in Ireland asking for dietary supplements and prescribed medication listed evening primrose oil, garlic, and ginseng [8]. The study focused on supplements that caused interactions with prescribed drugs. Species not involved in potential interactions in the study population were not reported in the paper. Garcia-Alvarez et al. conducted a retrospective consumer survey in six European countries (Finland, Germany, Italy, Romania, Spain, and UK), asking for use of plant dietary supplements [7]. Herbal medicinal products were excluded. In this study, 2874 respondents reported to have administered at least one herbal product during the past 12 months. Due to differences in consumer preference and the legal status of the products in different countries, the authors found a huge number of species that were each taken by a small proportion of participants. The ten species mentioned most often (by at least 102 participants or 3.5%) were ginkgo, evening primrose, artichoke, ginseng, aloe vera, fennel, valerian, soybean, lemon balm, and echinacea. A more recent survey of plant food supplement use (excluding herbal medicines), among 1230 participants, was published by Jeurissen et al. [9]. They found that products of a great variety of species (>100) were used with echinacea (14.2%), ginkgo, cranberry, ginseng, algae (such as spirulina and chlorella), citrus bioflavonoids, grape seeds, valerian, rose hip, and garlic (4.7%) being the ten most frequently reported. As “algae” and “citrus bioflavonoids” are poorly defined, they were not included in the list.

Knotek et al. published data on herbal product use by Czech adults, obtained in 1000 face-to-face interviews [28]. They found a prevalence of 56.6%. The most frequently used plants were *Mentha x piperita* (18.4%), *Melissa officinalis* (12.4%), *Plantgo lanceolata* (12.2%), *Tilia cordata* (11.8%), and *Matricaria recutita* (10.7%).

Information about specific HMPs and herbal supplements used by patients with rheumatoid arthritis (RA), chronic inflammatory bowel diseases (IBD), or osteoarthritis are scarce. Most surveys on complementary and alternative therapy or supplement use by those patients did not ask for the species used [4,19,29] or were conducted outside of the geographical area of this review [30,31,32], or both [33,34].

Among surveys on supplement use by arthritis patients from the last 20 years, no survey from Europe that revealed actual plant species could be found. Thus, two studies from Canada and the USA were screened to find plants that patients might use. A study among 1063 Canadian rheumatology patients, 382 (35.9%) reported the use of “natural health products”, including minerals, vitamins, and glucosamine. The only plant listed as a joint-specific product was turmeric. It was used by 12 patients, or 3.1% of supplement users [32]. Skiba et al. conducted a survey in southern Arizona (USA) that was completed by 696 adult patients diagnosed with rheumatoid arthritis [30]. The most popular plants used concomitantly with prescribed therapy were turmeric (43.5% ever usage, 30.3% current usage), ginger (25.5%/13.3%), and flaxseed (25%/9%). About 10% reported experience with boswellia or milk thistle, respectively.

Usage of complementary therapies, including herbal products by patients with IBD (Crohn’s disease, ulcerative colitis), is widespread in Europe [4,19,29,35]. However, most surveys did not ask for the plant species used. A study among children <18 years and their guardians in Scotland, with 86 patients who completed the survey, reported aloe vera, garlic, echinacea, and evening primrose [36]. Garlic and echinacea are probably taken for other purposes. *Boswellia serrata* was mentioned in two surveys among adults in Germany, which do not specify other herbal products [19,35]. A Spanish study reports *Aloe vera* [37].

The only indication to be found in HMPC monographs that fits the diseases used as example, while being of acceptable specificity, is “relief of minor articular pain”. Herbal drugs that can claim traditional use against this symptom are meadowsweet (herb and flowers), ash leaf, devil’s claw root, bogbean leaf, blackcurrant leaf, willow bark, and nettle herb. Patients with osteoarthritis or rheumatoid arthritis could decide to try products based on these herbal drugs.

There are positive clinical data for the complementary use of *Plantago ovata* [38], curcumin [39], and a combination of myrrh, chamomile, and coffee charcoal [40] by patients with ulcerative colitis, preferably as approved herbal medicinal product.

Wormwood extract can reduce the amount of steroids needed by Crohn’s disease patients [41,42]. The extract is not marketed, but patients might try to use tea or tinctures.

Cannabis flowers have been used by patients with various diseases to treat pain, appetite loss, and muscle spasms. The drug and preparations of it (e.g., oil extracts) gain popularity among RA [43], osteoarthritis [44], and IBD [45] patients. Animal studies and in vitro experiments suggest immune modulatory and anti-inflammatory properties of cannabinoids, which makes them possible candidates for therapy of autoimmune diseases (reviewed in [46]). All plants considered in this review are summarized in Table 1.

### 3.2. Potential Herb–Drug Interactions

Common drugs for the treatment of rheumatoid arthritis, IBD and osteoarthritis were taken from surveys [30,36] and treatment guidelines [23,24,25,47,48,49]. Table 2 shows the drugs considered with their indications and interaction potential.

Long-term therapy of RA and IBD consists of immune modulating drugs, such as methotrexate, azathioprine, 5-aminosalicylates, janus kinase inhibitors (tofacitinib and baricitinib), and a growing number of biologicals. The significance of JAK inhibitors is rising, and approvals of several drugs for chronic inflammatory/autoimmune diseases, especially RA, can be expected over the coming years. For RA, also hydroxychloroquine, chloroquine, and leflunomid are used. In both RA and IBD, cyclosporine is an option if first line therapies fail. During therapy establishment (RA) or acute episodes, glucocorticoids are applied to quell inflammation. RA patients use cyclooxygenase inhibitors (non-steroidal antirheumatic drugs, NSAIDs) episodically to treat articular pain, until their newly started or disease modifying therapy reaches full potency. Acetaminophen is less effective than NSAIDs and can be considered for patients with contraindications. In rare cases, when no other options for symptomatic pain therapy are left, opioids may be used.

Pharmacokinetic interactions with mAbs and other therapeutic proteins are rare [50]. Exceptions among therapeutic proteins are alterations of CYP expression, as a side effect of signaling modification. The most prominent example is tocilizumab, an IL-6-receptor antagonist, which elevates CYP 1A2, 2C9, 2C19, and 3A4 expression [50]. More important, biologicals carry the risk of immune reactions, from mild skin reactions to anaphylaxis. Another problem is loss of function, due to anti-mAb-antibodies. Additionally, there are target-specific risks, the most important for the immune modulating biologicals used in therapy of chronic inflammatory diseases being elevated infection risk and reactivation of inapparent infections [51].

Some biologicals (abatacept and tocilizumab) may cause elevated liver enzymes and are linked to rare cases of clinical DILI. Liver injury is more common with TNFα-inhibitors, such as infliximab [26]. Methotrexate, leflunomide, and acetaminophen pose a risk of hepatotoxicity, as well [52]. Combination of these drugs with liver toxic herbs should be avoided. Many potentially hepatotoxic herbs are not studied extensively enough to determine a safe daily dose.

Patients suffering from osteoarthritis are mainly treated non-pharmacologically, but episodic or long-term use of pain medication is widespread, with NSAIDs as the most common group. They can be combined with proton pump inhibitors (PPI) to protect against gastrointestinal ulceration and bleeding. Chronic pain can also be treated with selective serotonin and noradrenaline reuptake inhibitors (SSNRI), such as duloxetine. Short-term use of opioids for patients who cannot reach pain relief otherwise may occur, e.g., to bridge the time until surgical intervention.

For many herbal drugs, no interactions are known, or there are only scarce or contradicting in vitro data. This is indicated in Table 3 that summarizes the interaction potential of all plants considered in this review, with emphasis on the chosen plants and patient population. Selected herbals are discussed below.

#### 3.2.1. Aloe Vera Gel

*Aloe barbadensis* leaves contain a gel rich in mono- and polysaccharides that is used in cosmetics and as food flavor. It is suspected to have anti-inflammatory properties. For alopolysaccharide, a polymer from aloe gel, JAK2 inhibition was confirmed in vitro and in a rat model [111]. However, clinical data supporting oral use to treat diseases (e.g., ulcerative colitis) are scarce.

No clinical relevant interactions have been reported for aloe vera gel. Yet, the leaf rind latex (aloe juice) contains anthraquinones (aloin and derivatives), which are considered potential carcinogens [112]. They act as laxatives and thus can induce hypokalemia with chronic use. A study with rhubarb (*Rheum palmatum*) anthraquinones in rats suggests the inhibition of multidrug resistance protein 2 (MRP2) and decreased methotrexate excretion [113]. Whole leaf extracts and gel that was contaminated with latex during harvest contain anthraquinones. To avoid anthraquionone-associated risks, product quality is of great importance if patients wish to try aloe vera gel.

There are case reports of liver injury caused by aloe vera. One case names 30 mL aloe vera gel per day [114], while, for others, it is not stated whether the product contained gel or whole leaf extract [115,116]. One patient took 420 mg extract per day over three months [115]. Another patient experienced liver injury after taking 500 mg leaf powder every 2–3 days as a laxative [117]. In relation to other plants, such as green tea, HILI seems to be rare with aloe vera [118].

A cell culture study suggests that aloe vera gel can open tight junctions between intestinal epithel cells and, thus, enhance drug absorption [119], but clinical data are missing.

Aloe vera gel has a very low potential for interactions. Patients taking prescription drugs that are potentially hepatotoxic, such as methotrexate or leflonomid, should be careful or avoid aloe products. It is to be considered that there is no sufficient clinical evidence for the benefits of oral use of aloe vera gel to treat inflammatory diseases. There are only food supplements available, with insufficiently defined product quality.

#### 3.2.2. Cannabis

With the legalization of medical cannabis products in a growing number of countries, the use of this plant and its extracts to treat symptoms of chronic diseases increases. Still, a significant proportion of patients obtains the marijuana they intend to use medically from illegal sources with unknown quality (e.g., 52.9% of cannabis users among IBD patients in a survey conducted 2019 in Germany [45]). Besides, cannabidiol (CBD), a non-psychotropic cannabinoid, gained popularity and is used as an ingredient in cosmetics and marketed as a food supplement. This is not yet covered by European legislation, as these products would have to be approved as “novel food”, with proof of safety.

There are several chemotypes with great variation in cannabinoid content and composition. The best studied and, to current knowledge, most important components are Δ^9^tetrahydrocannabinol (THC), responsible for the psychotropic activities of cannabis, and CBD. CBD seems to diminish the adverse effects of THC, and synergism between the various derivatives found in plants and extracts has been postulated [120,121]. The plant is used to treat pain and appetite loss and receives interest as anti-inflammatory agent.

Cannabinoid receptors are mainly located in the brain (CB1) and on immune cells (CB2) (reviewed in [46]). Additionally, cannabinoids bind, to different extents, to several other receptors, including 5-HT2 and 3, M1, M4, glutamate receptors, and vanilloid-1 receptor, which is present in nociceptive neurons [46]. Cannabinoids affect T- and B-cells via CB2R and, thus, act immune modulatory. They lower IL-17 and γ-IFN secretion and increase IL-10 secretion, among further effects on immune cells and their behavior [46]. Additionally, CBD targets TNF-activated rheumatoid arthritis synovial fibroblasts in vitro and could delete proinflammatory immune cells and synovial fibroblasts, which would also explain the anti-inflammatory effects in rheuma models [122]. The authors postulate that there may be synergistic effects with methotrexate or JAK inhibitors. Animal model experiments, surveys, and small pilot studies show potential benefits in IBD treatment over short periods (8 weeks), while longer use (6 months) is associated with a higher rate of surgery (reviewed in [123]).

Cannabis, and its extracts, show a high potential for interactions. Common side effects are drowsiness, somnolence, and euphoria [60], which are intensified in combination with other CNS depressant drugs. The combination of cannabinoids and opioids should be avoided. Antagonism at muscarinic receptors may cause tachycardia, when combined with other anticholinergic drugs.

Cannabinoids are substrates of CYP3A4 and 2C9. In vitro data suggest the influence of cannabinoids and their metabolites on several CYP 450 enzymes [62,124]. The strongest effects were CYP2C9, 2B6, and 2D6 inhibition, but CYP 1A2, 2C19, and 3A4 might be affected as well. The prescription information of Sativex, a cannabis extract preparation approved for symptomatic treatment of multiple sclerosis patients, warns that CYP3A4, 1A2, 2B6, 2C9, and 2C19 are inhibited, and the mRNA expression of CYP1A2, 2B6, and CYP3A4 is induced [60]. Clinical outcomes of such opposing effects are difficult to estimate, and in vitro data concerning CYP 3A4, 1A2, and 2C19 are contradictory. Patients receiving medication affected by the mentioned enzymes should be monitored, especially in case of drugs with a small therapeutic window, such as cyclosporine and tacrolimus. This is supported by a case report of a patient on 5 mg (twice daily) tacrolimus, who received CBD (titrated to 20 mg/kg/d) in an epilepsy study and experienced elevated tacrolimus plasma concentrations and signs of toxicity [63]. Cannabinoids inhibit UDP-glucuronosyltransferase (UGT) 1A6, 1A9, 2B4, and 2B7 [125]. This can affect drugs, such as propofol, morphine, acetaminophen, and ibuprofen.

Cannabis preparations are emerging as a promising option for co-treatment of inflammatory diseases. There is much research needed to determine which patients benefit and the optimal dose and composition, as well as treatment time. Treatment with cannabinoids has to be carefully considered because of the high interaction potential. For many potential interactions, the clinical relevance is unknown, and differences between extracts and chemotypes are possible. Patients should be educated and carefully monitored. A special problem is that patients may try to self-medicate with illegally obtained cannabis flowers, which brings quality issues and makes it even more likely that they will not reveal that treatment to their health professional.

#### 3.2.3. Devil’s Claw

The roots of this plant, originating from southern Africa, are traditionally used to treat digestive disorders and mild articular pain [126]. Aqueous extracts have been shown to have anti-inflammatory and antioxidant effects. There are promising clinical data for the use in treatment of osteoarthritis. For the treatment of IBD, there have been ex vivo experiments showing positive results, but no clinical studies, so far (effects reviewed by Menghini et al. [127]). Inhibition of cyclooxygenase 2 seems to contribute to the anti-inflammatory effect [128]. Adverse effects in clinical studies are rare and mostly restricted to digestive complaints and allergies [129].

There are only in vitro data concerning possible pharmacokinetic interactions. Romiti et al. described pGP inhibition in renal cells [130], which might result in elevated serum levels of cyclosporine and glucocorticoids [131]. The extent was different for the three commercial products. Harpagosid did not inhibit the transport protein. After three days of treatment, pGP expression increased [130]. Elevated gene expression may balance protein inhibition to a certain degree, but might result in higher clearance after stopping Harpagophytum. The clinical significance of these in vitro findings is unclear.

Several in vitro studies found weak inhibition of CYP enzymes, especially CYP3A4 [96,97,132]. Interestingly, Modarai et al. found CYP3A4 inhibition by five out of ten commercial products, but not by harpagoside and harpagid [132]. There were different results for different batches of the same product. It seems that unidentified and, thus, unquantified substances are responsible for this effect.

Overall, the potential for clinical relevant interactions with devil’s claw seems to be low. Patients taking cyclosporine should be monitored when starting or discontinuing Harpagophytum preparates. Patients with stomach or duodenal ulcers should avoid Devil’s claw because of the COX-1 inhibition and content of bittering agents [126,128,133].

#### 3.2.4. Echinacea

Herb and roots of several *Echinacea* species are common for prevention and treatment of common cold. Patients also take the plant for general strengthening of the immune system. Best studied are root extracts of *Echinacea purpurea*, preferably with standardized alkylamide content [134]. Because of the immune stimulating effect, echinacea products are contraindicated for patients with autoimmune diseases or immune modulating therapy, based on theoretical consideration. This is debated, and there are hints to possible beneficial effects [135]. However, as long as clinical data are missing, co-use of echinacea and immune modulating drugs should be avoided.

There are conflicting data regarding possible pharmacokinetic interactions. There are reports of inhibition of CYP 3A4 [136,137], as well as induction of CYP 3A4 expression [88], possibly via activation of pregnaneX-receptor (which also induces CYP 1A2 and MDR1) [138]. Gorski et al. found opposite effects on intestinal and hepatic CYP 3A4 in a small clinical repeated measures design study (12 volunteers, 8 days, application of probe drugs, before and after echinacea), possibly because of differences in expression induction [139]. There are also in vitro [140,141] and open label repeated measure designed clinical studies [142,143] that find no effects. Considering their wide use, standardized Echinacea root extracts are considered safe, regarding pharmacokinetic interactions [104,135,144]. Still, possible effects, due to individual susceptibility, should be taken into account for patients receiving drugs with highly CYP 3A4-dependent metabolism.

#### 3.2.5. Evening Primrose Oil

Evening primrose oil is used to treat systemic inflammatory diseases and promote women’s health [145], but clinical evidence is scarce. The popular oral use against eczema has been proven ineffective in a meta-analysis [146]. There have been case reports of seizures with evening primrose oil [147] and animal studies suggesting platelet inhibition [148,149]. The doses applied in the rabbit study were relatively high (90, 180, and 360 microl/kg/d, corresponding to 5.4/10.8/21.6 mL/d or 5.0/10.0/20 g/d for 60 kg humans) [149]. A small randomized clinical trial with RA patients did not find differences in platelet activity between evening primrose oil, omega-3 oil, and fish oil supplementation [150], but these oils’ effects on platelet aggregation are debated, as well. Recently, thrombocytopenia, after hysterectomy in a patient chronically taking evening primrose oil and black seed oil (*Nigella sativa*), was reported [151]. Nigella sativa oil contains thymoquionone, which can lead to thrombocytopenia. The authors discuss the case as multifactorial, with the surgery and both herbal oils contributing to the development of thrombocytopenia [151]. Overall, the clinical risk of evening primrose oil seems to be low, but cumulative thrombocyte inhibitory effects of prescription drugs and combined herbal treatments should be taken into regard.

#### 3.2.6. Garlic

An herbal drug widely used to prevent atherosclerosis and treat common cold is garlic [152,153]. The European pharmacopoeia monographs pulverized dried bulbs of *Allium sativum* containing at least 0.45% allicin [154]. Clinical data suggest favorable influence of garlic on risk factors for atherosclerosis, such as blood pressure and hypercholesterolemia (reviewed in [152]). Garlic also shows possible antimicrobial properties (reviewed in [155]). Active constituents are organic sulfur components, the best studied of which are alliin and allicin, which are derived from alliin by the enzyme alliinase [156].

Garlic is available in a great variety of preparations. There are tablets and capsules, with garlic powder or dry extract prepared with ethanol-water mixtures, as well as oily extracts and distilled garlic oil. The latter contains numerous volatile allyl sulfides, but not alliin or allicin [157]. Allicin has been suspected to be the main active compound; due to its instability, enteric coated products containing alliin and alliinase were used to enable allicin formation in the intestine, but bioavailability varies between products and study data were contradictory [158]. It has been shown that, instead, water-soluble sulfur compounds, such as S-allylcysteine, which can be obtained by prolonged extraction with ethanol-water mixtures (aged garlic extract), are probably responsible for the desirable effects, while lacking the toxic potential and odor of allicin and its products (reviewed in [159]).

There have been small open label clinical studies to determine possible influences on metabolizing enzymes and pGP, usually via probe drugs. Markowitz et al. supplemented 14 volunteers, for 14 days, with 1800 mg dry extract, containing alliin equivalent to 1800 µg allicin twice daily. They did not observe changes in CYP 2D6 or 3A4 activity [71]. Gurley et al. measured 22% inhibition of CYP 2E1 but no influence on 2D6 or 3A4 after 28 days of supplementation with 500 mg garlic oil three times daily in twelve elderly (60–76 years) volunteers [70]. Hajda et al. supplemented ten volunteers with 600 mg garlic extract (yielding 3600 µg allicin) daily for 21 days and observed no influence on the expression of CYP 3A4 but an increase of intestinal pGP expression to 131% [72]. Based on this limited evidence, garlic preparations seem to be safe concerning CYP metabolism, but caution is recommended with pGP substrates.

Case reports of bleeding complications after excessive garlic ingestion raised concerns regarding the bleeding risk of patients taking garlic preparations [160,161]. Beckert et al. studied a platelet inhibition via a PAF-100 assay in ten healthy volunteers. After baseline measurement, they received 1000 mg garlic powder per day (there was no information available concerning the thiosulfinate content for the commercial product used) for two weeks. No significant difference to the baseline measurement was observed. After a washout period, ASA was tested as a positive control [73]. Mohammed et al. conducted a small crossover study in ten healthy volunteers, in order to detect a possible interaction with warfarin. They received a single dose of 5 mg, with or without two weeks pretreatment with a commercial product containing 2000 mg fresh garlic bulb, equivalent to 3.71 mg allicin, which was continued one week after warfarin application. Blood samples were drawn at several time points before and after warfarin application and INR, warfarin concentration, platelet aggregation, and Factor II, VII and X activity were determined. No difference was observed between garlic treatment and control periods [74]. Macan et al. compared 5 mL aged garlic extract (standardized to S-allylcysteine) against the placebo in parallel design in 48 participants on warfarin treatment for 12 weeks. No INR change or bleeding events were observed [162].

Clinical data on possible interactions of garlic products are limited due to the wide variety of tested products that makes studies difficult to compare. Considering the mostly negative results and the widespread use of garlic as medicinal plant, as well as food with few reports of potentially dangerous incidents, garlic bears a low-risk for interactions. Patients taking cyclosporine should be monitored when starting or ending garlic supplementation because of the possible induction of intestinal pGP.

#### 3.2.7. Ginkgo

Medicinal products based on ginkgo are used to treat (vascular) dementia, vertigo, and peripheral arterial disease. There are products containing a dry extract (DER 35-67:1, acetone 60% m/m; EGb 761) with regular market authorization for these applications [163]. The HMPC-monograph classifies this extract as “well established use” for “the improvement of (age-associated) cognitive impairment and of quality of life in mild dementia”. Besides the well-documented and -controlled medicinal products, there are food supplements that are made of various extracts or powdered leaves. The latter may contain elevated amounts of ginkgolic acids, which are potent allergens and possibly express cytotoxic, genotoxic and carcinogenic properties [164]. For the refined quantified dry extract, the ginkgolic acid content is limited to 5 ppm [154].

Ginkgo was discussed to induce CYP3A4 after reducing midazolam plasma levels in a repeated measures design clinical study [165], but there are other small clinical trials that did not find differences in CYP3A4 activity [70,166,167]. A recent meta-analysis found no effect of ginkgo on CYP3A4 [168].

Based on cases of severe bleeding events associated with ginkgo [169,170], the plant has been suspected to increase bleeding risk of patients with anticoagulant or platelet inhibiting co-medication. Inhibition of platelet aggregation factor (PAF) by ginkgo leaf ingredients has been discussed controversially [171]. There are in vitro experiments confirming thrombin inhibition [172]. Yet clinical studies [81,173] and a meta-analysis [174] could not find an elevated bleeding risk or influence on coagulation parameters. A small randomized, double-blind, placebo-controlled clinical study, with patients taking 325 mg/d ASA plus EGb 761 or placebo, found no difference in platelet function or bleeding/ bruising reports [175]. In a study analyzing a medical database that included several thousand patients using warfarin, with or without ginkgo, an elevated bleeding risk was found for concomitant use. Despite excluding patients whose ginkgo use was recorded at the same time as the bleeding event, there still may be a reporting bias. No information was provided about dosage and other medication [176]. In conclusion, a possible elevation of bleeding risk by ginkgo seems to be small. Therapy monitoring is advisable for high-risk patients, as well as discontinuing ginkgo before planned surgery.

#### 3.2.8. Ginseng

Ginseng is among the most popular herbs and applied by healthy, mostly elder persons, as well as patients with chronical diseases (3.1., Table 1). The drug has a long tradition as “tonic” or adaptogen, a substance that is meant to enhance physical and mental performance or help regain strength, in East Asia [177] and has been adopted into western herbal medicine.

The term “ginseng” can be used for several drugs. Most often it refers to *Panax ginseng* (Korean ginseng) roots. They can be differentiated into dried (white ginseng) and steamed (red ginseng), but this distinction is not always made. The monograph “ginseng radix” in the European Pharmacopoeia names only *P. ginseng* and defines a minimum content of 4.0% ginsenosides (triterpene saponins) [154]. Siberian ginseng, *Eleutherococcus senticosus*, is a different plant from the same family (Araliaceae), which contains different triterpene saponines (eleutherosides) and is used for similar applications. It is sometimes mixed up with *Panax*-species, but has its own PhEur monograph (“Eleutherococci radix”). It is used in Europe, but less popular than *P. ginseng* [7,9]. *Panax quinquefolium* (American ginseng) and *P. notoginseng* (Chinese ginseng) are more important in North America and East Asia.

There have been plenty of in vitro, in vivo and mostly small clinical studies regarding the effects of ginseng extracts or ginsenosides on CYP enzymes and transport proteins. Results are contradictory. Gurley et al. found no influence on CYP3A4, 1A2, and 2E1 and a slight (7%) inhibition of CYP2D6 in healthy volunteers [70]. Other clinical studies found induction (1000 mg extract/d) [178] or weak inhibition (2.1 g dried root/d) [179] of CYP3A4. A case report of imatinib-induced liver toxicity promoted by ginseng in energy drinks speculates about CYP3A4 inhibition as potential interaction mechanism [180]. Seong et al. could not confirm any influence of red ginseng extract on CYP2C9, 3A4, 1A2, 2C19, 2D6, and OATP1B1 in a repeated measures probe cocktail study with fifteen healthy volunteers [181]. There may be inter-individual differences in susceptibility. Hao et al. showed in cell culture experiments that ginsenosides and their deglucosylated metabolites show different effects on CYP1A1, 1A2, and 3A4 [182]. If that is the case in vivo, interindividual differences in enzyme activity could contribute to the interaction potential.

A case report of liver injury in a patient applying atorvastatin and Siberian ginseng points to possible CYP3A4 and/or OATP1B1 inhibition by *Eleutherococcus senticosus* extracts [183].

Ginseng has been discussed as to promote bleeding. A small double-blind, randomized study with patients with cardiac valve replacement found no difference of INR between warfarin + ginseng and warfarin + placebo [184]. In a randomized, assessor-blinded study with 42 participants Wang et al. found that ASA + *P. notoginseng* extract results in stronger platelet inhibition than ASA + placebo, but reduced gastric mucosa injury [185]. Lau et al. compared the influence of extracts of the three *Panax*-species on in vitro platelet aggregation and rat bleeding time and found the effect strongest in *P. notoginseng* and weakest in *P. quinquefolium* [186]. Kwon et al. stated, based on in vitro experiments, that the composition of ginsenosides seems to be more important for the effect on platelet inhibition than the total content [187]. Ginsenosides and ASA mutually enhance their absorption in Caco-2 cells and rats [188,189], but there are no clinical data regarding this phenomenon. A study investigating platelet aggregation with P. ginseng alone and with ASA on 25 volunteers found that P. ginseng did not increase ASA’s inhibitory effect on platelet aggregation [190].

For *P. quinquefolium*, a small study with healthy volunteers showed a reduced anticoagulant effect of warfarin and lower plasma concentrations [191]. The mechanism is unknown.

As ginseng may lower blood glucose and is studied as potential antidiabetic [192], it might increase the hypoglycemia risk by chloroquine or hydroxychloroquine. The clinical relevance is probably low [193]. Patients should know and pay attention to the symptoms.

Overall, the potential of ginseng for herb–drug interactions is low. Caution is advisable for patients who take ASA and have additional risk factors for bleeding events, as well as patients who apply CYP3A4 substrates with a small therapeutic window. Therapy should be monitored if eleutherococcus is combined with CYP3A4 substrates, especially drugs with a narrow therapeutic window, such as cyclosporine.

#### 3.2.9. Green Tea

The intake of beverages prepared from green tea (unfermented leaves of *Camellia sinensis*), has been part of food culture in East Asia for centuries. In modern times, it has gained popularity worldwide. Besides the traditional preparation of infuses and use as flavor, a vast market of food supplements that contain leaf powder or, more often, various extracts (GTE) has grown. Tea has been suggested to have health benefits in prevention of cardiovascular disease, cancer and neurodegenerative diseases (reviewed in [194]) and is part of supplements advertised for weight control. Clinical evidence is still sketchy. The leaves contain flavonoids and catechins, the most active and most abundant of which is (-)-epigallocatechin-3-gallate (EGCG). Dey et al. recommend in their review on beverages and nutrition of RA patients moderate tea consumption because of antiinflammatory effects of catechins and caffeine [195].

Green Tea has been associated with HILI. A scientific opinion of EFSA [196] from 2018 states that the common food consumption is safe, as there are only few cases of HILI associated with tea consumption despite the widespread use. However, the regular application of green tea extracts as food supplements, with doses above 800 mg EGCG per day, is associated with an increase of serum transaminases that can indicate liver injury. The risk is higher when the supplement is taken under fasting conditions and/or as single bolus instead of split doses due to higher EGCG bioavailability. The Minnesota Green Tea Trial assessed the safety of GTE supplementation for one year in postmenopausal women with elevated risk of breast cancer [197,198]. In this randomized, double-blind study, 513 participants were treated with GTE (mean daily dose 1315 mg total catechins, 843 mg EGCG, split into two doses) and 508 with placebo. In the verum arm, 5.1% of participants developed moderate to severe liver function abnormalities. Serum ALT returned to normal when GTE was discontinued and raised again upon rechallenge [198]. The authors stress that women drinking more than seven alcoholic beverages per week or with BMI >40 were excluded from the study. HILI could occur more frequently in these groups because of the combination of risk factors. In conclusion, GTE supplementation, especially with high doses, should be avoided by patients with elevated risk of liver injury or hepatotoxic medication, such as methotrexate or azathioprine. Moderate tea consumption is less problematic regarding HILI. It still may cause liver injury in rare cases due to individual susceptibility and should be assessed in unclear cases of liver impairment.

Interestingly, GTE showed protective effects against acetaminophen-induced hepatotoxicity in mice when given before the drug, but increased acetaminophen toxicity when given after [199,200].

Green tea has been suspected to antagonize Warfarin, but evidence is scarce and contradicting [201,202]. Patients taking coumarin derivatives should be monitored when changing GTE supplementation or pronounced tea drinking habits.

The effect of GTE and EGCG on various cytochrome P 450 enzymes has been studied in vitro and in vivo. Green tea extract and EGCG inhibited CYP2B6, 2C8 and 3A4 in pooled liver and intestine microsomes. The isoenzymes 2C19 and 2D6 were inhibited, as well, but with IC50 48.7 and 25.1 µg/mL, which the authors argue is above what can be expected in vivo [203]. Another study found that green tea influences flurbiprofen (probe drug for CYP2C9) in vitro, but not in a crossover study with 14 volunteers; but they only tested drinking ~240 mL brewed green tea after flurbiprofen application, neither high amounts nor longer use [202]. Yao et al. applied atorvastatin in rats after 3 weeks of drinking green tea or water. In the tea group, the AUC of atorvastatin (+85%) and several metabolites were elevated. They found intestinal CYP3A4 activity to be lowered, and hepatic CYP3A4 activity increased. OATP2 expression was decreased, which could have led to limited hepatic uptake [204].

Clinical studies showed no [205] or minor [206] effects on CYP3A4 activity and no effect on CYP1A2, 2D6 and 2C9. There is a case report of elevated serum levels of tacrolimus in a renal transplant patient after green tea consumption. The concentration returned to normal with GT abstinence. Genotyping revealed the patient as “poor metabolizer” with alleles CYP3A4*1B and CYP3A4*10. The authors state that CYP3A4 inhibition or pGP inhibition could have caused the interaction. They favor CYP, as the patient could be unusually susceptible due to the individual genotype [207].

A bigger issue than CYP interactions is the inhibition of several transport proteins by green tea catechins. GT catechins inhibit pGP in vitro [208,209] and in vivo. A small clinical repeated measures design study (n = 15) was done with digoxin as probe drug. It revealed reduced Cmax and AUC (0.69/0.72) under GTE supplementation (single dose as well as 28 d pretreatment, 630 mg/d) with high interindividual variability [210].

Cell culture studies show inhibition of folate uptake by EGCG and GTE [211] that extends to methotrexate [212,213], as well as inhibition of dihydrofolate reductase by EGCG [214]. Clinical data on folate uptake-inhibition are rare and contradictory: Alemdaroglu et al. conducted a small (7 healthy volunteers) open-label randomized cross-over study which investigated the intake of 0.4 and 5 mg folic acid with green tea versus water [215]. Cmax was reduced by 39.2% (0.4 mg) and 27.4% (5 mg), AUC was reduced by 26.6% and 39.9%. Test subjects took 250 mL GT or water three times daily two days before the test day and five times during the test day (30 min before, with and until 2 h after folic acid). With 0.3 g extract/250 mL (207.7 µmol/g EGCG in the extract) this corresponds to 900 and 1500 mg extract/ day with 85.6/142.8 mg/d EGCG. Augustin et al. compared 670 mg GTE (81.74 mg EGCG)/day versus placebo over 3 weeks in a randomized, double-blind parallel study with 31 participants [216]. They observed no difference in the folate serum concentration. The daily catechin dose in this study was slightly lower and the folate intake of the participants was less strictly controlled than in the first trial. Theoretically, MTX absorption could be impaired by GT catechins and/or adverse effects could be promoted by folate deficiency. Yet, clinical data are lacking, especially concerning the question of how long GT inhibits the folic acid transporter and if a certain amount of waiting time between GT and MTX or folate intake could solve the problem.

Green tea inhibits OATP1A2 (organic anion transporting polypeptide), as has been demonstrated in vitro [209] and in small clinical crossover studies [217,218]. The absorption of the OATP1A2-substrate nadolol was reduced (decrease of Cmax and AUC by 85%, reduced blood pressure lowering effect) without alterations of tmax, half-life and clearance. The inhibition of uptake transporters persists at least one hour after green tea consumption [219]. Additionally, in vitro studies show inhibition of OATP1B1, 2B1, and 1B3 [209,220].

Main concern regarding herb–drug interactions with green tea is the inhibition of OATPs, especially OATP1A2, which can affect absorption, liver-uptake (1B, 2B) or renal excretion (1A2) [221]. Influence on hepatic or renal transport proteins requires sufficient absorption of catechins and such requires high doses of GTE. Application on empty stomach increases bioavailability of GT catechins and hence the risk of interactions and adverse effects. The inhibition of pGP or CYP3A4 could be relevant in susceptible individuals, especially when combined with substrate drugs with small therapeutic index. Methotrexate, leflunomide, azathioprine, cyclosporine and tacrolimus should not be combined with green tea extracts or high amounts of green tea. Dietary supplements with their often insufficient specification and declaration should generally be avoided.

Interestingly, Malaviya reported symptom control in 55% of 542 RA patients with MTX intolerance in northern India by drinking coffee on the day of MTX application [222]. This was explained by the author with caffeine acting as adenosine receptor antagonist and thus counteract MTX-induced adenosine release. Regarding the possible absorption inhibition and liver toxicity risk of high amounts of EGCG, coffee or black tea would be better nutritional caffeine sources for patients taking MTX than green tea.

#### 3.2.10. Tumeric

Tumeric (*Curcuma longa*) and its main active substance curcumin show anti-inflammatory activity addressing multiple targets [223,224] and there is a growing body of evidence for benefits in inflammatory diseases [225,226] Still, there is a need for more high quality clinical studies and addressing the poor bioavailability [227,228]. Contribution of modulating effects on the gut microbiome is also discussed [229].

Curcuminoids have been shown to inhibit CY3A4 and 2C9 in vitro [230]. A small (n = 6) repeated measures design study with dextromethorphan as probe drug showed inhibition of CYP2D6 and minor inhibition of CYP3A4 by 2 × 1.5 g/d [231]. Rats treated with 18 mg/kg *C. xanthorrhiza*-extract showed a higher AUC of warfarin compared to the controls [232]. This could have been caused by CYP2C9 inhibition. Additionally, sulfotransferase and glutathione transferase were inhibited in vitro, which might amplify acetaminophene toxicity [230,233]. The clinical relevance of these data is unclear.

Turmeric extract did not inhibit platelet function alone or in combination with acetyl salicylic acid in a crossover study with 25 volunteers [190].

In conclusion, turmeric and curcumin preparations seem to be safe for most patients, but combination with CYP3A4, 2C9, and 2D6 substrates with low therapeutic index should be monitored.

#### 3.2.11. Willow Bark and Meadowsweet Herb/Flowers

Both drugs contain flavonoids and salicylates and are traditionally used to treat fever and pain. Salicylates might increase the effects of NSAIDs on coagulation as well as their gastro- and nephrotoxicity. Willow bark extract showed a smaller effect on platelet aggregation in humans than ASA [234]. Ibuprofen and other cyclooxygenase inhibitors inhibit the cardioprotective platelet inhibitory effect of low dose ASA when applied before ASA [235]. If this interaction occurs as well with salicylic acid derivatives in plant extracts is not known.

Extract doses applied in clinical studies contain low amounts of salicin (240 mg/d). Other substances, especially flavonoids, are suspected to be relevant for the effect [236]. Willow bark extract is effective in treatment of lower back pain (low to moderate evidence) [237], but a study with OA and RA patients was negative [238]. The authors state that the reason could be a different composition of the extract compared with the previous pilot study due to a switch from *Salix purpurea x daphnoides* to the more commonly used *S. daphnoides*. They found differences in the content of other substances aside of salicin, which was standardized.

There are small clinical studies that suggest reduced renal elimination of methotrexate, when combined with high doses of ASA [239], magnesium trisalicylate, ibuprofen, and naproxen [240]. With MTX doses between 7.5 and 15 mg weekly, this effect is moderate (AUC +28% with ASA, less for the others), and there is inter-individual variability. Salicylates from herbal drugs might show similar effects. The clinical significance of this phenomenon depends on individual susceptibility.

The combination of herbal drugs that contain salicylates with NSAIDs, in doses used for pain treatment, should be avoided. Patients taking methotrexate should be cautious and discontinue the herbal product if adverse effects increase.

## 4. Discussion

### 4.1. Reasons for Uncommon Interactions and Adverse Effects

Medicinal plants and plant extracts are complex mixtures of chemical substances and, as such, can address multiple targets. Metabolite contents vary depending on plant species, chemotype, growth conditions, harvest, and processing, as well as the extraction process. Availability and use of differently prepared extracts results in barely comparable products and data. The predictability of HMP efficacy and possible adverse effects is constrained for many herbal drugs by the lack of knowledge, concerning their targets and active substances, as well as in the effective dose. Synergistic effects between several metabolites in plant extracts are suspected [241,242,243], but good clinical evidence is missing. Besides, the metabolites responsible for herb–drug interactions or adverse drug reactions need not be the same that produce the desirable effects. If available, standardized or quantified extracts should be preferred. According the HMPC, standardized herbal substances are adjusted to a given content of constituents with known therapeutic activity, whereas quantified herbal substances are adjusted to a given content range of active markers (substances that are generally accepted to contribute to the therapeutic activity). Herbal products with unknown active principles are defined solely by the production process. Analytical markers can be specified, but are not part of the declaration [244].

For plant derived products that are marketed as food supplements, quality requirements and declaration rules are less extensive. Manufacturers may prefer marketing their products as food supplements, instead of medicinal products, for safe development and production costs. However, these products are less reliable and cannot be recommended for the (adjuvant) treatment of diseases or non-desirable health conditions.

Important contributions of unknown substances to effect or interaction mechanisms can lead to fluctuations in effect strength between similar products or even batches of the same product, as mentioned for ginseng, willow bark, and devil’s claw. Considering the wide use of herbal products, these fluctuations are usually beneath clinical relevance. Still, caution should be taken in susceptible individuals (e.g., taking interaction sensitive prescription drugs, known CYP mutations). Much work remains in order to better understand mechanisms of desired and adverse effects of most medical herbs and the responsible constituents.

The interpretation of in vitro data regarding the influence of plant extracts on metabolizing enzymes or transporters is challenging, more so if single constituents are tested. Multiple effects can balance, as seems to be the case with CYP3A4 inhibition and induction by echinacea root extracts [139]. Not all activity changes that can be measured in vitro need to be clinically relevant. It is common that findings from in vitro studies, which seem alarming at first, cannot be confirmed in humans, sometimes documented in the same publication [202,245]. Possible reasons for this phenomenon can be found in the complex nature of plant extracts and are reviewed in [246]. In brief, it is difficult to estimate relevant test concentrations that reflect in vivo conditions, as knowledge on absorption (pre-absorption), metabolism, and the eventual active metabolite and plasma binding rates of the multiple substances is usually scarce. The extract constituents may influence each other, which makes studies with purified substances less representative and can cause reproducibility problems, due to natural variability, as seen in [132]. Nevertheless, in vitro studies are a fast way to discover potential concerns for further investigation and a necessary means to elucidate the underlying mechanisms and responsible constituents [246].

A potential risk of using herbal products from “unofficial” sources is adulteration with prescription drugs [247]. This has been reported most often for products advertised for weight loss, sexual performance, and sports performance enhancement and can be assumed to be less frequent in other products. It is still worth considering, should unusual effects occur.

### 4.2. The Patient’s Side

Patient’s reasons to use plant products of various qualities for medical purposes are diverse. They include short-term treatment of mild to moderate acute symptoms with HMPs or herbal teas and long-term intake of supplements or HMPs for specific conditions or general well-being.

Patients with chronic diseases, who use complementary therapies, including herbal products, hope to optimize their therapy, reduce conventional drugs (especially steroids), or mitigate adverse effects of their prescribed therapy. Besides that, emotional well-being, taking responsibility for their therapy, and the wish for a holistic approach are frequent motivations [10,19,248]. In this setting, it can be reasonable for a patient to take a product with insufficient evidence of efficacy, as a means of self-empowerment, sufficient quality, and safety, in combination with the prescribed therapy provided.

It is important to make patients aware of the fact that plants can cause adverse effects and are not safe in every situation by being “natural”. Many patients talk about complementary therapies, including herbal products, only if asked directly, often because they think the products are harmless and information not important for their health professional [5,249,250,251,252]. Another reason is fear of a negative reaction [5,251]. This puts emphasis on an open and trustful practitioner–patient relationship. A majority of patients wish to be informed about complementary therapies by their physician [251]; however, in real life, the most important sources for information are friends, family, and media [28,248]. Respondents in Knotek et al.’s survey also frequently named pharmacists as source of information. This survey asks not only for dietary supplements or complementary therapies, in general, but for medicinal herbs, in general, and the most important health issues were cold and digestion problems [28]. There may be a difference in patient behavior, depending on the reason for using herbal products. Still, this stresses the responsibility of pharmacists, who are a possible source of herbal medicinal products, to raise awareness for possible risks and educate the patients. Pharmaceutical care programs that include medication assessments pose another opportunity to obtain a full account of the patient’s medication and supplementation and discover problematic combinations.

Practitioners should be aware that patients might use herbal products intermittently. In Garcia-Alvarez et al.’s consumer survey, 37.3% of respondents reported that they take their supplements “periodically” [7]. While regular, long-term use of herbals with kinetic interaction potential may contribute to titration or dose finding of newly started drugs and, thus, not affect the outcome, regular discontinuing and restarting of the plant product would be more likely to cause problems. Hence, it is reasonable to ask not only for current use of herbal products but for temporary, repeated use, as well.

### 4.3. Limitations

Most of the surveys used to determinate frequently used plants focused on dietary supplements and excluded herbal medical products. This excludes plants represented exclusively or predominantly as HMPs in the respective countries, as well as herbal teas. The legal status of products with the same plant may differ largely between countries. Valerian, for example, is marketed in Germany, mainly as HMP, and, thus, possibly a great proportion of consumption was not included in the survey by Garcia-Alvarez [7]. In many countries, food supplements and HMPs can be found of popular plants. Elsewhere, the same plant can be found only as a supplement or HMP. Detailed information about the legal status of valerian, St. John’s wort, ginseng, green tea, and ginkgo in European countries are provided in [13].

Especially plants commonly used to treat symptoms of common cold are likely underrepresented (such as thyme, primrose, ivy, licorice, and yellow gentian). Likewise, the list of frequently used plants in this article misses some plants for the treatment of digestive disorders (caraway, rosemary, anise, and cinnamon), possibly because they are rarely marketed as dietary supplements. Digestive problems occur widespread and are often treated without consulting a physician. Patients suffering from inflammatory bowel disease may try herbs in search for relief if they cannot achieve sufficient symptom control with their prescribed drugs.

Several countries, mainly in Middle and Eastern Europe, are not represented in the survey data that the plant selection was based on.

Plants are known by numerous common names in different countries, and sometimes the same name is used for more than one species. This may result in translation errors or assigning mentions to the wrong species, in some cases.

The majority of clinical studies on herb–drug interactions are conducted on small groups of healthy and younger volunteers. They are likely to miss interactions that depend on individual condition or susceptibility (e.g., gene variants and liver or renal impairment). This can explain contradictions between clinical trials and case reports (as for CYP2C9 inhibition by ginger or milk thistle, see Table 3). Gauging the risks for patients with complex diseases, based on such data, is challenging.

The focus of this survey lies on RA, IBD, and osteoarthritis, as well as the medication that patients are usually treated with. Interactions with drugs beyond this topic are sometimes mentioned (especially anticoagulants), but incomplete.

## 5. Conclusions

The use of herbal products for health improvement is common among patients. It is more frequent among women and elderly people. Among patients with chronic diseases, those with poor symptom control are more likely to try complementary/alternative medicines, including herbal products. Concomitant use with prescription medicine bears the risk of interactions. For many potential herb–drug interactions, the clinical significance is still discussed and high-quality data are rare. Reasons for individual susceptibility are often unknown. The following points should be considered to handle the matter:

- The basis is a trusting and respectful relationship between patient and health professional. Patients should not be afraid to admit interest in, or use of, alternative therapies, food supplements, or self-acquired medical products.
- Health professionals should explicitly ask about use of herbal products, sensitize patients for the importance of such information, and encourage them to seek advice for risk assessment before they try self-acquired medical products or supplements.
- Herbal medicinal products, preferably with standardized or quantified extracts, should be preferred over food supplements, as quality and declaration requirements are more extensive for medicinal products. Food supplements should not be actively recommended.
- A brief list of high- and low-risk plants can be found in Table 4.
- It is important to report suspected herb–drug interactions, in order to collect data. Information on patient, medication, the suspected product, and the course of treatment should be collected as complete as possible.

## Figures and Tables

**Table 1:** This table lists the plants considered for possible interactions, with both their English and botanical names. It indicates if they were reported in general surveys or by patients with rheumatoid arthritis/inflammatory bowel disease, if they could be considered useful for treatment of these conditions, and the references.

Plant	General Surveys		RA	IBD	HPMC *
	Common	Mentioned			
Ginkgo (*Ginkgo biloba*)	[7,9,27]				
Evening primrose oil (*Oenothera biennis*)	[7,8,27]	[9]		[36]	
Artichoke (*Cynara scolymus*)	[7]				
Ginseng (*Panax ginseng*) **	[7,8,9,27]				
Aloe vera gel (*Aloe barbadensis*)	[5,7,27]	[9]		[36,37]	
Fennel (*Foeniculum vulgare*)	[7]	[9]			
Valerian (*Valeriana officinalis*)	[7,9]				
Soybean (*Glycine max*)	[7]	[9]			
Lemon balm (*Melissa officinalis*)	[7,28]	[9]			
Echinacea (*Echinacea purpurea, E. angustifolia*)	[5,7,9,27]			[36]	
Garlic (*Allium sativum*)	[5,8,9,27]	[7]		[36]	
St. John’s wort (*Hypericum* *perforatum*)	[27]	[7,9,28]			
Devil’s claw (*Harpagophytum* *procumbens, H. zeyheri*)	[27]	[7,9]			+
Cranberry (*Vaccinium* *macrocarpon*)	[5,9,27]				
Saw palmetto (*Serenoa repens*)	[27]	[7,9]			
Bilberry (*Vaccinium myrtillus*)	[5]	[7,9]			
Green tea (*Camellia sinensis*)	[5]	[7,9]			
Ginger (*Zingiber officinale*)	[5]	[7,9]	[30]		
Grape seeds (*Vitis vinifera*)	[9]	[7]			
Rose hip (*Rosa canina*)	[9]	[7,28]			
Peppermint (*Mentha x piperita*)	[28]	[7,9]			
English plantain (*Plantago lanceolata*)	[28]	[7,9]			
Lime flowers/linden flowers (*Tilia cordata*)	[28]	[7]			
Chamomile (*Matricaria recutita; M. chamomilla*)	[28]	[7,9]			
Turmeric (*Curcuma longa*)		[7,9]	[30,32]	[39]	
Flaxseed (*Linum* *ussitatissimum*)		[7,9]	[30]		
Indian frankincense *Boswellia serrata*		[7]	[30]		
Milk thistle (*Silybum marianum*)		[7,9]	[30]		
Meadowsweet (*Filipendula ulmaria*)		[7]			+
Ash leaf (*Fraxinus excelsior*)		[7]			+
Bogbean leaf (*Menyanthes trifoliate*)					+
Blackcurrant leaf (*Ribes nigrum*)		[7,9]			+
Willow bark (*Salix purpurea, S. daphnoides, S. fragilis*)		[7]			+
Nettle herb (*Urtica dioica, U. urens*)		[7,9,28]			+
Psyllium (*Plantago ovata*)		[7,9]		[38]	
Myrrh, chamomile, coffee charcoal				[40]	
Wormwood (*Artemisia absithium*)		[7]		[41]	
*Cannabis sativa*			[30,43]	[45]	

* HMPC monograph stating “Traditional herbal medicinal product for the relief of minor articular pain”; ** most often Korean ginseng (*Panax ginseng*), but may be confused with American ginseng (*Panax quinquefolium*) or Siberian ginseng (*Eleutherococcus senticosus*).

**Table 2:** Conventional drugs in therapy of RA, IBD, or osteoarthritis and their interaction potential (focus on mechanisms that can influence the effect of the drug or elevate its risks, not on their potential to influence others).

Drug/Group	Application	Possible Interactions and Risks
Methotrexate (MTX)	RA, IBD	Hepatotoxic; mainly renal elimination (OATP1A2); OATP1B 1/3 substrate
Leflunomide	RA	Hepatotoxic; moderately induces CYP1A2, 2C8, inhibits OATP1B1
5-aminosalicylates	RA, IBD	nephrotoxic
Chloroquine, Hydroxychloroquine	RA	QT-prolongation, hypoglycemia when combined with blood glucose lowering drugs
Azathioprine	RA, IBD	hepatotoxic
Calcineurin inhibitors	RA, IBD	Cyclosporine: CYP3A4, OATP1B1/1B3, and pGP substrate
	IBD	Tacrolimus: CYP3A4 and pGP substrate
JAK-inhibitors	RA	Baricitinib: OAT1/3 substrate
	RA, IBD	Tofacitinib: CYP3A4 substrate
Glucocorticoids	RA, IBD	CYP3A4 substrates: budesonide, dexamethasone, betamethasone, prednisolone, prednisone, methylprednisolone, hydrocortisone, deflazacort; pGP-substrates: budesonide, dexamethasone, hydrocortisone, prednisolone, methylprednisolone elevated risk of GI bleedings with NSAIDs
NSAIDs	OA, RA	Platelet inhibition (esp. acetyl salicylic acid (ASA), excl. COX-2 selective subst.), reduced renal perfusion, GI toxicity, cardiovascular risk (esp. COX-2 selective subst.) CYP2C9 substrates (diclofenac, ibuprofen) CYP3A4 substrate: etoricoxib
(Acetaminophen)	RA, OA	hepatotoxic
PPI	(OA, RA)	Omeprazol: substrate of CYP2C19, 3A4
(Opioids)	RA, OA, IBD	Many CYP2D6 and pGP substrates, CNS depression
SSNRI	OA	Duloxetine: CYP1A2 substrate

**Table 3:** This table shows the plants/herbal combination products included in this review, in alphabetic order, and possible herb–drug interaction mechanisms and risks that could cumulate. The right column lists drugs applied in RA/osteoarthritis/IBD treatment that are, or could, be affected.

Plant	Drug Interactions/Risks	Drugs Affected
Aloe vera gel	Depends on product quality; see 3.2.1.	
Artichoke	-	-
Ash leaf	-	-
Bilberry	Anthocyanins have been discussed as platelet aggregation inhibitors [53]; decreased platelet activation in metabolic syndrome patients [54]	(NSAIDs)
Blackcurrant leaf	-	-
Bogbean leaf	No interactions known (contraindication: gastric or duodenal ulcer) [55]	-
Boswellia	Unspecific CYP450 inhibition [56] and transport protein modulation (OATP1B3, MRP2, pGP) in vitro [57,58]; two case reports of elevated INR in warfarin patients [59] (possibly by CYP interaction)	Caution with cyclosporine and tacrolimus
*Cannabis sativa*	Increases central nervous system (CNS) depression [60]	Opioids, SSNRI (e.g., duloxetine)
	Inhibition of UGT1A9 and UGT2B7 [60]; CYP3A4 and 2C9 substrate, possible influence on CYP1A2 [61] (induction by THC, induction or inhibition by CBD)	Duloxetine (several drugs in other fields; propofol, anticoagulants!)
	in vitro: inhibition of several CYP enzymes by cannabinoids and main metabolites, including CYP 2B6, 2C9, 2D6; minor inhibition: 1A2, 2C19, 3A4 [60,62]	Several drugs, including opioids, NSAIDs and possibly cyclosporine and tacrolimus (CBD) [63]
	anticholinergic agents (risk of tachycardia)	(several drugs in other fields)
Chamomile	Minor CYP3A4 inhibition in vitro [64]; case reports of elevated cyclosporine serum level [65,66]	Cyclosporine
Cranberry	Case reports of potentiated warfarin effect; clinical studies: no difference [67]	
	One case report of lowered tacrolimus serum concentration [68]	Tacrolimus
Devil’s claw	Possible CYP3A4 inhibition (in vitro data only)	Cyclosporine
Echinacea	May diminish therapeutic effect of immunosuppressants	Methotrexate, leflunomide, azathioprine, biologicals, JAK- inhibitors, cyclosporine, tacrolimus, systemic glucocorticoids
	Possible influence on CYP3A4	cyclosporine
English plantain	-	-
Evening primrose oil	Possible inhibition of platelet aggregation	NSAIDs
Fennel	-	-
Flaxseed	Can delay or reduce drug absorption; 1 h time-lag between application [69]	Minerals, vitamins, drugs
	obstruction risk with drugs that inhibit peristaltic movements	Opioids
Garlic	Inhibition of CYP2E1 [70], but not 2D6 and 3A4 [70,71]; induces pGP [72]	Cyclosporine, tacrolimus
	Elevated bleeding risk due to platelet inhibition suspected [53,73]; contradicting clinical data [73,74]	Monitor patients on anticoagulants when starting/ending garlic preparations; caution with antiplatelet drugs (NSAIDs, especially ASA)
Ginger	Contradicting data regarding CYP2C9, 3A4, and pGP inhibition in vitro [75,76,77]; cases of interactions with dabigatran (pGP) [78], phenprocoumon (CYP2C9) [79], and crizotinib (CYP3A4, 2C9; pGP) [80]; no effect in a clinical study with warfarin (CYP2C9) [81]; elevated tacrolimus AUC in rats [82]	CYP2C9, 3A4 and pGP substrates with narrow therapeutic window, such as cyclosporine, tacrolimus
	Inhibits platelet aggregation in vitro [83]	Caution with anticoagulant and platelet-aggregation inhibiting drugs (NSAIDs)
Ginkgo	Possible inhibition of platelet aggregation	NSAIDs, especially ASA
Ginseng	Possible inhibition of platelet aggregation (conflicting data)	(NSAIDs, especially ASA)
	Possible CYP3A4 inhibition	cyclosporine, tacrolimus
	Possible blood glucose lowering effect	chloroquine, hydroxychloroquine
Grape seeds	[84,85]	-
Green tea	Inhibition of OATP1A2, 1B3, 2B1, pGB; risk of liver injury; possible CYP3A4 inhibition	Methotrexate, leflunomide, azathioprine, cyclosporine, tacrolimus
Lemon balm	-	-
Lime/linden flowers	-	-
Meadowsweet	Contains salicylates (possibly elevated bleeding risk and GI injury with NSAIDs; may reduce renal elimination)	NSAIDs, glucocorticoids; 5- aminosalicylates; may reduce clearance of methotrexate
Milk thistle	Inhibits UDP1A6 in vitro [86]; CYP3A4 and 2C9 inhibition suspected, but no relevant influence in small clinical studies [87,88,89,90,91]; case report of warfarin interaction, probably due to CYP2C9 inhibition [92]	Caution with CYP3A4 and 2C9 substrates with small therapeutic windows (such as cyclosporine)
Myrrh, chamomile, coffee charcoal	Can impair absorption of simultaneously applied drugs [93]	Caution with cyclosporine
	minor CYP3A4 inhibition in vitro [64]; case reports of elevates cyclosporine serum level [65,66] (chamomile) Myrrh: CYP3A4 induction in vitro [94]	
Nettle herb	-	-
Peppermint	Peppermint tea inhibits CYP3A4 induction by rifampicin in vitro [95]; oil: contradicting results on CYP inhibition in vitro [96,97]; enhanced cyclosporine bioavailability in rats [98]	Cyclosporine, tacrolimus?
Psyllium	Can delay or reduce drug absorption; 1 h time-lag between application [99]	Vitamins, minerals, drugs prednisolone/ fludrocortisone [100]
Rose hip	-	-
Saw palmetto	Case report bleeding [101]; most literature argues against interaction [102,103,104]	(Warfarin?)
Soybean	in vitro: no relevant effect on CYP2D6 and 3A4 [64,77,105]	-
St. John’s wort	Induces CYP2C9, 2C19, 3A4; pGP [106] Serotonin syndrome [107]	Cyclosporine, tacrolimus, tofacitinib, glucocorticoids, omeprazole, opioids (and many more in other fields), SSNRI
Turmeric	Possible inhibition of CYP2D6, 2C9, 3A4 Elevated AUC of tacrolimus in rats [82]	Caution with cyclosporine, tacrolimus, coumarins
	Inhibition of sulfotransferase and glutathione transferase	Acetaminophen
Valerian	Increases CNS depression	Opioids, SSNRI (e.g., duloxetine)
	No CYP1A2, 2D6, 2E1, 3A4 interactions found [108,109,110]	
Willow bark	Contains salicylates (possibly elevated bleeding risk and GI injury with NSAIDs; may reduce renal elimination)	NSAIDs, glucocorticoids; 5- aminosalicylats; may reduce clearance of methotrexate; (warfarin)
Wormwood	-	-

**Table 4:** Short summary of the interaction risk of the reviewed plants in RA, IBD, and osteoarthritis treatment. Details can be found in Table 3 and Section 3.2.

Interaction Potential	Plants
High interaction potential, may affect several drugs	St John’s wort, cannabis (3.2.2.), green tea (3.2.9.), echinacea (3.2.4.)
Moderate interaction potential; few drugs or lower level of evidence	Flax seed, ginger, meadowsweet, psyllium, valerian, willow bark (3.2.11.)
Generally low interaction potential, but possible interaction with cyclosporine/tacrolimus via CYP or pGP (case reports or in vitro data; Table 3)	Boswellia, chamomile, cranberry, devil’s claw (3.2.3.), garlic (3.2.6.), ginseng (3.2.7.), milk thistle, peppermint, turmeric (3.2.10.)
No interactions reported/very low-risk	Artichoke, ash leaf, blackcurrant leaf, English plantain, fennel, lemon balm, lime/linden flowers, nettle herb, rose hip, saw palmetto, soybean, wormwood

## Data Availability

Not applicable.
